# Immunological Reaction in TNF-**α**-Mediated Osteoclast Formation and Bone Resorption *In Vitro* and *In Vivo*

**DOI:** 10.1155/2013/181849

**Published:** 2013-05-23

**Authors:** Hideki Kitaura, Keisuke Kimura, Masahiko Ishida, Haruka Kohara, Masako Yoshimatsu, Teruko Takano-Yamamoto

**Affiliations:** ^1^Division of Orthodontics and Dentofacial Orthopedics, Department of Translational Medicine, Tohoku University Graduate School of Dentistry, 4-1 Seiryo-machi, Aoba-ku, Sendai 980-8575, Japan; ^2^Department of Orthodontics and Dentofacial Orthopedics, Nagasaki University Graduate School of Biomedical Sciences, 1-7-1 Sakamoto, Nagasaki 852-8588, Japan

## Abstract

Tumor necrosis factor-**α** (TNF-**α**) is a cytokine produced by monocytes, macrophages, and T cells and is induced by pathogens, endotoxins, or related substances. TNF-**α** may play a key role in bone metabolism and is important in inflammatory bone diseases such as rheumatoid arthritis. Cells directly involved in osteoclastogenesis include macrophages, which are osteoclast precursor cells, osteoblasts, or stromal cells. These cells express receptor activator of NF-**κ**B ligand (RANKL) to induce osteoclastogenesis, and T cells, which secrete RANKL, promote osteoclastogenesis during inflammation. Elucidating the detailed effects of TNF-**α** on bone metabolism may enable the identification of therapeutic targets that can efficiently suppress bone destruction in inflammatory bone diseases. TNF-**α** is considered to act by directly increasing RANK expression in macrophages and by increasing RANKL in stromal cells. Inflammatory cytokines such as interleukin- (IL-) 12, IL-18, and interferon-**γ** (IFN-**γ**) strongly inhibit osteoclast formation. IL-12, IL-18, and IFN-**γ** induce apoptosis in bone marrow cells treated with TNF-**α** *in vitro*, and osteoclastogenesis is inhibited by the interactions of TNF-**α**-induced Fas and Fas ligand induced by IL-12, IL-18, and IFN-**γ**. This review describes and discusses the role of cells concerned with osteoclast formation and immunological reactions in TNF-**α**-mediated osteoclastogenesis *in vitro* and *in vivo*.

## 1. Introduction

Tumor necrosis factor-*α* (TNF-*α*) plays a major role in host defense, and it exerts proinflammatory activities through various cells including mononuclear phagocytes, where it is responsible for the activation of cytocidal systems [1]. TNF-*α*-induced osteoclast recruitment is probably central to the pathogenesis of disorders involving inflammation [2]. Osteoclasts are multinucleated giant cells formed by the fusion of precursor cells of the monocyte/macrophage lineage, which originate from hematopoietic stem cells and are uniquely responsible for *in vivo* bone resorption [3]. Bone destruction is marked in rheumatoid arthritis, a disease characterized by proliferative synovitis in which proteases secreted by the synovial membrane cause cartilaginous inflammation leading to joint destruction [4]. This bone destruction is caused by inflammation-induced osteoclasts. TNF-*α*, produced by cells within the articular tissue, causes inflammation by inducing synovial cell proliferation, promoting inflammatory cytokine production, and increasing vascular endothelial cell permeability [5]. Furthermore, TNF-*α* causes osteoclast-induced bone destruction as well as the inhibition of osteoblast differentiation and apoptosis [6]. TNF-*α* acts on chondrocytes and induces the synthesis of proteases such as collagenase and matrix metalloproteinase, which cause cartilage destruction [7]. Therefore, TNF-*α*-targeting biological drugs are effective for treating rheumatoid arthritis [8].

## 2. Osteoclastogenesis and TNF-***α***

In 1998, two different research groups noted that receptor activator of NF-*κ*B ligand (RANKL) was essential for osteoclast differentiation [9, 10]. RANKL induces osteoclast differentiation by binding to RANK, a membrane-binding protein expressed on the surface of macrophage-colony-stimulating-factor- (M-CSF-) induced osteoclast precursors derived from myeloid cells and monocytes [11]. TNF-*α* was also reported to induce the formation of osteoclastic cells from bone marrow macrophages *in vitro* [12, 13]. Alternatively, in the presence of TNF-*α*, osteoclast formation is induced by low concentrations of RANKL, and TNF-*α* increases the effects of RANKL. In the absence of RANKL, osteoclast differentiation does not occur [14]. Thus, TNF-*α* is considered to enhance RANKL signaling. Subsequently, TNF transgenic (TNF-Tg) mice × RANK^−/−^ mice were created, and lack of RANK resulted in no changes in osteopetrosis even in the presence of TNF-*α*. This suggested that TNF-*α*-induced osteoclastogenesis is dependent on RANKL [15]. However, when M-CSF induces precursor cell formation from myeloid cells in the presence of transforming growth factor-*β* (TGF-*β*), osteoclastogenesis is induced by TNF-*α* alone. Furthermore, TNF-*α* could induce osteoclast differentiation in osteoclast lacking mouse models, including RANKL^−/−^, RANK^−/−^, and TNF receptor-associated factor-6 (TRAF6)^−/−^ mice [16]. This suggested that TNF-*α*-induced osteoclastogenesis is independent of RANKL. Further studies are necessary to clarify these events. 

## 3. Analysis of TNF-***α***-Mediated Osteoclastogenesis **In Vivo**

**Figure 1:**
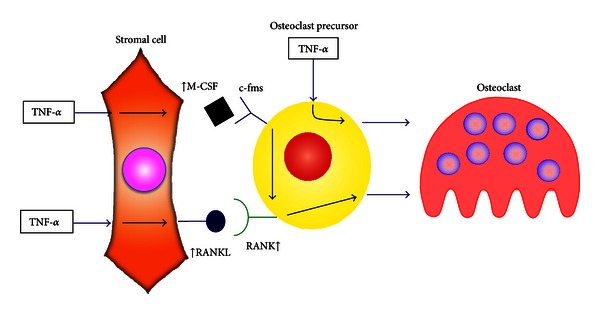
Contribution of macrophage and stromal cell in TNF-*α*-mediated osteoclastogenesis. TNF-*α* stimulated expression of RANKL and M-CSF in stromal cell, and the stromal cell induced osteoclastogenesis. Also, TNF-*α* directly induced osteoclastogenesis to osteoclast precursor in the presence of constitutive level of RANKL and TNF-*α*-induced M-CSF stimulate expression of RANK in osteoclast precursor.

Cells directly involved in osteoclastogenesis include macrophages, stromal cells that express RANKL and induce osteoclastogenesis, and T cells that express RANKL and promote osteoclastogenesis [17, 18]. TNF-*α* plays a central role in inflammatory osteoclastogenesis. Therefore, a better understanding of the contribution of these target cells *in vivo* may provide important therapeutic implications. It was reported that macrophages are direct targets of TNF-*α in vitro* [14]. Teitelbaum's group analyzed the function of these cells in TNF-*α*-induced osteoclastogenesis *in vivo *using bone marrow transplants to determine the *in vivo* contribution of each cell type and whether they were a direct or indirect target of TNF-*α*. When a lethal dose of radiation was administered to mice, hematopoietic cells including macrophages were destroyed, but stromal cells survived. Donor myeloid cells were then transplanted into the irradiated recipients. The resultant chimeric mice contained recipient-derived stromal cells and donor-derived macrophages. Using this technique, 4 types of chimeric mice were created using wild type (WT) and both 55 kDa TNF receptor-1 (TNFR1) and 75 kDa TNF receptor-2 (TNFR2) deficient mice (KO). Each irradiated mouse underwent bone marrow transplant, and 4 groups were prepared as follows: (1) WT marrow transplanted into WT mice as a positive control for the administration of TNF-*α*, (2) WT marrow transplanted into KO, (3) KO marrow transplanted into WT, and (4) KO marrow transplanted into KO transplants as a negative control. Thus, groups of mice contained both TNFRs-bearing macrophages and stromal cells, TNFRs-bearing macrophages alone, TNFRs-bearing stromal cells alone, and those with TNFRs-deficient macrophages and stromal cells. After the marrow transplant, recipient T cells were blocked with anti-CD4 and anti-CD8 antibodies. These mice were injected with TNF-*α* in the calvariae and examined for osteoclastogenesis. The number of osteoclasts in KO to WT was less than that in WT to WT transplants. This suggested that TNF-*α* directly induced bone macrophages to undergo osteoclast differentiation. However, the osteoclast numbers in KO to WT were greater than those in WT to KO transplants indicating that while both bone marrow macrophages and stromal cells participate as direct cytokine targets in TNF-*α*-induced osteoclastogenesis, stromal cells are dominant. Analysis of RANKL and RANK expression by myeloid cells demonstrated that RANKL expression was increased in the WT to WT and KO to WT transplants. Thus, TNF-*α* may act directly on stromal cells to increase RANKL expression. Expression of RANK increased in the WT to WT, WT to KO, and KO to WT transplants. RANK expression also increased in mice with TNFRs-deficient macrophages. Considering these results, TNF-*α* may act by directly influencing macrophages to increase RANK expression and inducing stromal cells to increase expression of RANKL [19, 20]. TNF-*α* increases the number of bone marrow macrophages in WT to WT and KO to WT but not WT to KO transplants. M-CSF increases the proliferation of macrophages. Expression of M-CSF also increased in WT to WT and KO to WT transplants, suggesting that M-CSF was expressed in response to TNF-*α* produced by stromal cells. Therefore, TNF-*α*-induced M-CSF may increase the number of bone marrow macrophages. TNF-*α* directly increased RANK expression by influencing macrophages. Furthermore, the expression of RANK also increased in KO to WT transplants as described earlier. In addition, it was reported that RANK expression increased *in vitro* when macrophages were incubated with M-SCF. Thus, TNF-*α* may induce RANK expression via TNF-*α*-induced M-CSF from stromal cells. It was suggested that M-CSF also plays an important role in TNF-*α*-induced osteoclastogenesis* in vivo* [20] (Figure 1).

## 4. Signaling Pathways Associated with Osteoclast Differentiation

RANK, a receptor of RANKL, is expressed in osteoclast precursors. Mitogen-activated protein kinase (MAPK) pathways such as TNF receptor-associated factor (TRAF), family-mediated c-jun N-terminal kinase (JNK), p38, and nuclear factor-kappa B (NF-*κ*B) are activated by RANKL–RANK-induced stimulation [21]. Moreover, the activator protein 1 (AP-1) family, such as c-fos, is also activated. These molecules have been analyzed using various gene knockout mice. Two reports using TRAF6 deficient mice indicated osteopetrosis-like symptoms in both [22, 23]. Furthermore, osteopetrosis-like symptoms observed in NF-*κ*B and c-Fos deficient mice suggest that these molecules are essential for osteoclastogenesis [24–26]. Osteoclast differentiation is promoted by the transcription factors AP-1 and NF-*κ*B, which activate nuclear factor of activated T-cell c1 (NFATc1), the master transcription factor in osteoclast differentiation. NFATc1 is required for osteoclast differentiation [27] and is activated by calcineurin-mediated dephosphorylation, a nuclear calcium-dependent phosphatase [28]. NFATc1 migrates into the nucleus and fuses to upstream tartrate-resistant acid phosphatase (TRAP), an osteoclast specific gene, cathepsin K, calcitonin receptor, and osteoclast-associated receptor (OSCAR), thus promoting transcription [27]. Immunoreceptors such as OSCARs activate NFATc1, bind to adaptor molecules with immunoreceptor tyrosine-based activation motifs (ITAMS) expressed in osteoclast precursors, and function as costimulatory signals for RANKL [29]. M-CSF is an essential factor in osteoclast differentiation and a survival factor for osteoclasts. Osteoclast precursors express c-Fms, an M-CSF receptor, and M-CSF stimulation activates MAPK pathways such as extracellular signal-regulated kinase (ERK), phosphoinositide 3-kinase (P13 K), and Akt [11].

## 5. TNF-***α*** Signaling Pathways 

It was reported that TRAF6-deficient mice develop osteopetrosis with defects in bone remodeling caused by impaired osteoclast formation. TNF-*α* also required TRAF6 for osteoclast formation *in vitro* [30]. However, TRAF6 is not a common adaptor protein of TNFRs. Thus, TNF-*α*-induced osteoclast formation might be necessary for the existence of RANKL. However, *in vitro* experiments using fetal liver cells from TRAF2-deficient mice demonstrated that TNF-*α*-induced osteoclast formation was severely impaired. Therefore, TRAF2 may play an important role in TNF-*α*-induced osteoclast formation. Furthermore, RANKL-induced osteoclast formation was reduced in progenitors from TRAF2-deficient mice [31]. These studies indicate that TRAF2 signaling enhances RANK-TRAF6 signaling for osteoclast formation. It was reported that TNF-*α* and RANKL synergistically induce osteoclast formation [14]. It may indicate that TNF-*α*-induced TRAF2 signaling enhances RANKL signaling for osteoclast formation. TNF-*α* can induce biological reactions by either TNFR1 or TNFR2. Each receptor can mediate distinct intracellular signals. Analysis of the respective TNFR1 or TNFR2 deficient mice revealed that TNFR1 promotes osteoclast differentiation, whereas TNFR2 inhibits osteoclast differentiation [32]. The intracellular domain of TNFR1 is bound by an adaptor protein, TNF receptor-associated death domain (TRADD), which mobilizes additional adaptor protein receptor interacting protein-1 (RIP-1) and TRAF2 [33]. Subsequently, the TRADD-RIP-1-TRAF2 complex is released from TNFR1. The adapter proteins in the complex activate key signaling pathways. RIP-1 recruitment of MAPK extracellular signal-regulated kinase kinase 3 (MEKK-3) and TGF-*β*-activated kinase (TAK1) activates the I*κ*B kinase (IKK) complex. The IKK complex phosphorylates I*κ*B*α* that ubiquitinates and degrades I*κ*B*α*. This subsequently releases NF-*κ*B subunits, which translocate into the nucleus and promote gene transcription [34–36]. TNF-*α*-induced NF-*κ*B activation in macrophages can be mediated by c-Src, which is a nonreceptor tyrosine kinase [37]. Therefore, many signaling mediators can promote TNF-*α*-induced signaling pathways, depending on the cell type. Meanwhile, two types of TRAF-6-deficient mice have been reported. One contained only few weak TRAP-positive mononuclear cells [38]. Therefore, osteoclast formation is dependent upon TRAF6 signaling. However, the other TRAF-6-deficient mouse contained normal numbers of TRAP-positive osteoclasts [39]. Therefore, it may be possible that signaling other than TRAF6 is involved in osteoclast formation. The role of TNF-*α* signaling in osteoclastogenesis remains poorly understood, and further studies are required to elucidate the relationship between TNF-*α* and osteoclast differentiation.

## 6. The Role of TNF-***α*** in Osteoblast Function

Osteoblasts differentiate from mesenchymal stem cells. TNF-*α* has an inhibitory effect during various stages of osteoblast differentiation and can act on osteoblast precursor cells during the early stages of differentiation to inhibit insulin-like growth factor 1, which increases the differentiation of osteoblast precursor cells from stem cells [40]. Furthermore, TNF-*α* acts on osteoblasts to inhibit the transcription of runt-related transcription factor 2 (RUNX2), the master transcription factor for osteoblast differentiation, by promoting the degradation of RUNX2 mRNA [41]. TNF-*α* also inhibits MAPK-mediated osterix expression and promoter activity [6], increases Fas expression, and induces apoptosis. A previous study demonstrated that TNF-*α* increased IL-1 expression and IL-1-induced RANKL expression in bone-marrow-derived stromal cells, which promoted osteoclast differentiation [42].

## 7. Effect of Cytokines in TNF-***α***-Mediated Osteoclastogenesis and Bone Resorption

**Figure 2:**
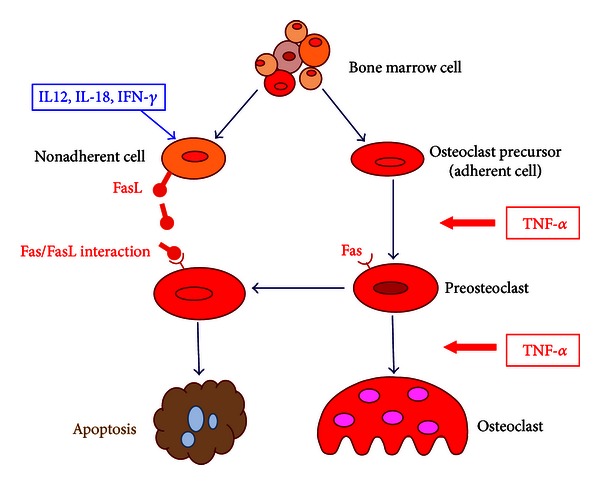
The mechanism of IL-12-, IL-18-, and IFN-*γ*-induced apoptosis in bone marrow cell culture. TNF-*α* induced the expression of Fas on preosteoclast, and IL-12 and IL-18 induce the expression of FasL on nonadherent cells. The Fas/FasL interaction induced the apoptosis of preosteoclast in bone marrow cell culture.

Inflammatory cytokines have multiple effects on bone resorption. *In vivo* immune and inflammatory responses are regulated by a complex network of cytokines. In rheumatoid arthritis, TNF-*α*, IL-1, IL-6, and IL-17 produced by synovial macrophages and T cells act on osteoblasts to promote RANKL expression [43]. Thus, IL-1 [42], IL-6 [44], and IL-17 [45], as well as TGF-*β* [46], promote osteoclastogenesis, whereas IL-4 [47–49], IL-10 [50], IL-12 [51–54], IL-13 [55], IL-18 [56–58], and IFN-*γ* [46] inhibit osteoclastogenesis. Furthermore, when IFN-*γ*, IL-12, and IL-18 are acted during TNF-*α*-mediated osteoclastogenesis in myeloid cells, TNF-*α* induces Fas expression and IFN-*γ*, IL-12, and IL-18 induce FasL expression leading to apoptosis of osteoclast precursors [53, 54, 57–59] (Figure 2). These results demonstrate that inhibitory cytokines may be applied clinically as inhibitors of joint destruction. However, the systemic administration of cytokines results in their poor localization in joints. Therefore, osteoclastogenesis can be experimentally inhibited by the overexpression of cytokine genes using a viral vector. Therefore, further studies are required to elucidate the pathology and cytokine-mediated regulatory mechanisms in bone metabolism. 

## 8. Role of TNF-***α*** in Joint Inflammation and Bone Destruction in Inflammatory Arthritis

TNF-*α* is the key mediator of joint inflammation and bone destruction in inflammatory arthritis, such as rheumatoid arthritis, psoriatic patients with arthritis, and juvenile idiopathic arthritis. Several studies have measured high amounts of TNF-*α* in the serum and synovial fluid of patients with rheumatoid arthritis and psoriatic arthritis and children with juvenile idiopathic arthritis [60–62]. Rheumatoid arthritis is an inflammatory disease caused by autoimmune responses, and it is aggravated by excessive bone resorption in the peripheral joints [63]. TNF-*α* is thought to play a crucial role in rheumatoid arthritis since it enhances the production of inflammatory disease-related molecules such as IL-1 or IL-6 in the serum and synovial fluid [64, 65]. TNF-*α* is sufficient to induce the development of all the symptoms of inflammatory arthritis when overexpressed in mice [66, 67]. Osteoclast precursors are increased in the peripheral blood of TNF-Tg mice and psoriatic patients with arthritis. This increase can be reversed by anti-TNF-*α* treatment [61, 68]. TNF-*α* increases marrow osteoclast precursor numbers in WT mice by promoting osteoclast precursor proliferation, differentiation and expression of the M-CSF receptor, c-Fms [69]. The relevance of TNF-*α* in human disease is underlined by the efficiency of TNF-*α* neutralizing therapy for the treatment of rheumatoid arthritis. Neutralization therapies using the soluble TNF receptor-2-IgG-Fc fusion protein, etanercept, or anti-TNF-*α* monoclonal antibodies such as infliximab have proved to be a successful strategy for ameliorating both inflammation and joint destruction in rheumatoid arthritis [70, 71]. However, TNF-*α*-targeting therapies have several disadvantages; for example, there is a risk of antidrug antibody production when using anti-TNF-*α* antibodies and they are expensive. Thus, there is a need to develop new drugs to neutralize TNF-*α*. TNF-*α* kinoid, a heterocomplex of human TNF-*α* and keyhole limpet hemocyanin (TNF-K), is an active immunotherapy targeting TNF-*α*. However, TNF-K induced anti-TNF antibody production [72]. The cyclic peptide WP9QY was designed to mimic the most critical TNF-*α* recognition loop on TNFRI, and it prevents the interactions of TNF-*α* with TNF receptors. The peptide inhibited osteoclast formation *in vitro* and *in vivo* in mice [73, 74]. 

## 9. Effect of M-CSF Blocking in TNF-***α***-Mediated Osteoclastogenesis and Bone Resorption

TNF-*α* plays a key role in inflammatory arthritis; TNF-*α*-targeting biological drugs are highly effective in the treatment of inflammatory arthritis. It was reported that administration of antibodies to c-Fms (anti-c-Fms antibody) completely blocked osteoclastogenesis and bone erosion induced by TNF-*α* administration. Furthermore, in the study on the efficacy of anti-c-Fms antibodies injected into the experimental arthritis model using the serum of K/B × N mice [75], there was no effect on the inflammatory cells; however, osteoclastogenesis was inhibited by the antibodies [20]. Lipopolysaccharide (LPS) is a major component of the cell wall of Gram-negative bacteria and is a potent inducer of inflammation and a pathogen of inflammatory bone loss [76–78]. LPS can induce the production of many local immune factors, including proinflammatory cytokines such as TNF-*α* and IL-1, from macrophages or other cells in inflammatory tissues [79]. These cytokines are associated with LPS-induced osteoclast formation and bone destruction in both *in vivo* and *in vitro* studies [80–82]. It was reported that anti-c-Fms antibody affects bacterial LPS-induced osteoclastogenesis and bone resorption, and also LPS induce expression of RANK *in vivo* [83]. With regard to the involvement of TNF-*α* in osteoclastogenesis in arthritis, M-CSF blocking may be effective. Although treatments targeting TNF-*α* are highly effective in inhibiting the progression of bone destruction caused by inflammatory arthritis, other cytokine inhibitors that effectively target bone destruction should be developed and considered for concomitant usage.

## 10. Conclusion

Many studies have indicated that TNF-*α* is a key molecule for inflammatory osteoclastogenesis and bone destruction during inflammatory arthritis. Furthermore, the role of cells involved in TNF-*α*-induced osteoclast formation and the interactions between TNF-*α*-induced osteoclast formation and cytokines and their signaling pathways have gradually become clear. Currently, TNF-*α* is the major target of highly effective biological drugs for the treatment of inflammatory arthritis. The progress of studies for TNF-*α*-induced osteoclast formation and bone destruction has been accelerated. However, there is still much to learn. In addition, TNF-*α*-targeting therapies have several drawbacks. Therefore, further studies are required to fully understand TNF-*α*-induced osteoclast formation and bone destruction.
